# Supplementary material for: Thinking about it: the impact of COVID-19-related stimuli on prospective memory
Source: PeerJ. 2023 Nov 16;11:e16389. doi: 10.7717/peerj.16389 (PMC10657566; doi:10.7717/peerj.16389)
Supplement: Supplemental Information 5 — Set of scales and questions composing the questionnaire participants responded to, partly before and partly after the task. [file peerj-11-16389-s005.pdf]

ITALIAN VERSION

I. Questionario – Parte 1

Demographics

Età (anni):

Genere:

- ○ Femmina
- ○ Maschio
- ○ Altro: ____

Titolo di studio:

Negli ultimi due anni, hai vissuto la maggior parte del tempo in Italia?

- ○ Si
- ○ No

Indica in che regione e in che provincia:

Regione

Provincia

Start of Block: Domande legate al benessere (ansia e depressione)

Hai mai avuto patologie di tipo neurologico (trauma cranico, ischemie) o psichiatrico (disturbi di personalità, depressione)?

- ○ Si, di tipo neurologico
- ○ Si, di tipo psichiatrico
- ○ No, mai
- ○ Si, entrambe

Specifica quale patologia:

- ☐ Ictus o cerebrolesioni acquisite
- ☐ Morbo di Parkinson
- ☐ Morbo di Alzheimer
- ☐ Sclerosi Multipla
- ☐ Altro (*specifica*): ____

Specifica quale patologia:

- ☐ Disturbi dell'umore (depressione, disturbo bipolare)
- ☐ Disturbi di personalità
- ☐ Disturbi d'ansia
- ☐ Disturbi ossessivo compulsivo
- ☐ Disturbi psicotici
- ☐ Disturbi alimentari
- ☐ Altro (*specifica*): ____

Attualmente assumi psicofarmaci?

- ○ Si
- ○ No

Le seguenti domande si riferiscono al tuo stato di BENESSERE

ISTRUZIONI: Per favore, leggi ogni frase e indica con quale frequenza la situazione descritta si è verificata negli ultimi sette giorni. Tieni presente che non esistono risposte giuste o sbagliate. Non impiegare troppo tempo per rispondere a ciascuna affermazione, spesso la prima risposta è la più accurata.

	**Non mi è mai accaduto**	**Qualche volta**	**Con una certa frequenza**	**Quasi sempre**
1. Ho provato molta tensione e ho avuto difficoltà a recuperare uno stato di calma	○	○	○	○
2. Mi sono accorto/a di avere la bocca secca	○	○	○	○
3. Non riuscivo proprio a provare delle emozioni positive	○	○	○	○
4. Mi sono sentito/a molto in affanno con difficoltà a respirare (per es., respiro molto accelerato, sensazione di forte affanno in assenza di sforzo fisico)	○	○	○	○
5. Ho avuto un'estrema difficoltà nel cominciare quello che dovevo fare	○	○	○	○
6. Ho avuto la tendenza a reagire in maniera eccessiva alle situazioni	○	○	○	○

7. Ho avuto tremori (per es. alle mani)	○	○	○	○
8. Ho sentito che stavo impiegando molta energia nervosa	○	○	○	○
9. Ho temuto di trovarmi in situazioni in cui sarei potuto andare nel panico e rendermi ridicolo	○	○	○	○
10. Non vedevo nulla di buono nel mio futuro	○	○	○	○
11. Mi sono sentito/a stressato/a	○	○	○	○
12. Ho avuto difficoltà a rilassarmi	○	○	○	○
13. Mi sono sentito/a scoraggiato/a e depresso/a	○	○	○	○
14. Non riuscivo a tollerare per nulla eventi o situazioni che mi impedivano di portare avanti ciò che stavo facendo	○	○	○	○
15. Ho sentito di essere vicino ad avere un attacco di panico	○	○	○	○
16. Non c’era nulla che potesse darmi entusiasmo	○	○	○	○

17. Sentivo di valere poco come persona	○	○	○	○
18. Mi sono sentito/a piuttosto irritabile	○	○	○	○
19. Ho percepito distintamente il battito del mio cuore senza aver fatto uno sforzo fisico (per es., battito cardiaco accelerato o perdita di un battito)	○	○	○	○
20. Mi sono sentito/a spaventato/a senza ragione	○	○	○	○
21. Sentivo la vita priva di significato	○	○	○	○

II. TASK: Link to the Pavlovia Page

Start of Block: Link to the task (video inside)

Ti chiedo di inserire qui un codice alfanumerico (ID_Soggetto) col quale verrai identificato, in forma del tutto anonima. Il codice, che dovrai inserire anche all'inizio del compito che troverai al link sottostante, deve essere composto da: iniziali del tuo nome e cognome, età, genere col quale ti identifichi (inserisci M, F, o A per altro) e giorno del mese in cui stai svolgendo l'esperimento (es. Mario Rossi, età 23, sesso M, esperimento svolto il 3 ottobre: MR23M3).

Ecco il link per accedere all'esperimento:

https://run.pavlovia.org/Stefano_PhD/thai_nega/html

Ricorda, alla voce ID_Soggetto inserisci il codice che hai appena inserito! Quando hai terminato il compito, torna su questa schermata per qualche ultima domanda.

____

III. Questionario – Parte 2

Experience with the pandemic

Hai mai contratto il COVID-19?

○ SI

○ NO

Hai il COVID-19 in questo momento?

○ SI

○ NO

Quali sintomi hai/hai avuto associati al COVID-19? (*è possibile selezionare più risposte*)

- [ ] Febbre
- [ ] Tosse
- [ ] Difficoltà respiratorie
- [ ] Senso di stanchezza/ debolezza
- [ ] Nessuno di questi sintomi

Hai avuto altri sintomi relativi al COVID-19? (*è possibile selezionare più risposte*)

- ☐ Indolenzimento e dolori muscolari
- ☐ Congestione nasale
- ☐ Naso che cola
- ☐ Mal di gola
- ☐ Diarrea
- ☐ Difficoltà nel sentire gli odori
- ☐ Difficoltà nel sentire i gusti
- ☐ Maggiore o minore sensibilità tattile
- ☐ Difficoltà uditive
- ☐ Difficoltà visive (come “vedere doppio”, occhi arrossati/ secchi, lacrimazione frequente)
- ☐ Nessuno di questi sintomi

In che condizione ti trovi attualmente a seguito dell’emergenza sanitaria da COVID-19?

- ○ Nessuna restrizione (zona bianca)
- ○ Posso uscire di casa seppur con alcuni limiti/ restrizioni (zona gialla/arancione)
- ○ Posso uscire di casa solo se strettamente necessario (zona rossa)
- ○ Non posso uscire di casa (isolamento domiciliare totale/ quarantena)

Da quanti giorni sei in isolamento/ quarantena?

____

Quanto hai paura di essere contagiato dal COVID-19?

- Per nulla
- Poca
- Moderata
- Abbastanza
- Molta

Con che frequenza cerchi informazioni a riguardo?

- Mai
- Raramente
- Una volta alla settimana
- Più volte la settimana
- Ogni giorno

Conosci qualcuno che abbia contratto il COVID-19?

	SI	NO
Familiare/i o partner	○	○
Amico/i	○	○
Conoscente/i	○	○

Conosci qualcuno che sia deceduto a causa del COVID-19?

	SI	NO
Familiare/i o partner	○	○
Amico/i	○	○
Conoscente/i	○	○

Hai fatto il vaccino anti-COVID-19?

- ○ Si, ho completato il primo ciclo e ho ricevuto anche la terza dose
- ○ Si, ho completato il ciclo di vaccinazione (due dosi)
- ○ Si, ma non ho ancora completato il ciclo (una dose su due)
- ○ No, ma ho in programma di vaccinarmi
- ○ No, non ho intenzione di vaccinarmi

Le seguenti domande si riferiscono al tuo stato di BENESSERE

ISTRUZIONI: Facendo riferimento alla situazione attuale, caratterizzata dall'epidemia di COVID-19 e dalle misure di distanziamento sociale messe in atto per contenerla, indica come ti senti per ciascuna delle seguenti dimensioni.

	Per niente	Un po'	Moderatamente	Abbastanza	Estremamente
1. Avere pensieri ripetuti, inquietanti e indesiderati relativi a questa esperienza stressante	○	○	○	○	○
2. Avere sogni ripetuti e inquietanti relativi a questa esperienza stressante	○	○	○	○	○
3. Sentirsi molto turbato	○	○	○	○	○
4. Avere forti reazioni fisiche pensando a questa esperienza stressante (es. cuore martellante, difficoltà a respirare)	○	○	○	○	○
5. Cercare di evitare pensieri e sentimenti legati a questa esperienza stressante	○	○	○	○	○
6. Avere difficoltà a	○	○	○	○	○

pensare ad aspetti diversi da questa situazione stressante					
7. Avere difficoltà a concentrarti	○	○	○	○	○
8. Avere problemi ad addormentarti	○	○	○	○	○
9. Avere un sonno disturbato	○	○	○	○	○

Page Break

L'attuale pandemia ha modificato vari aspetti della nostra quotidianità. Come valuteresti l'impatto del COVID-19 sui seguenti aspetti della tua vita?
Dai una valutazione su una scala che va da 0 (nessun impatto) a 5 (molto impatto).

	Nessun impatto<br>0	1	2	3	4	Molto impatto<br>5
LAVORATIVO/ ECONOMICO						
SALUTE FISICA						
SALUTE PSICOLOGICA						
RELAZIONI FAMILIARI						
RELAZIONE SENTIMENTALE						
**RELAZIONI SOCIALI**						

Sei mai stato seguito a livello psicologico prima della pandemia?

- ○ SI
- ○ NO

Hai cercato un aiuto psicologico durante la pandemia?

- ○ SI
- ○ NO

Vorresti cercare un aiuto psicologico in futuro?

- ○ SI
- ○ NO

ENGLISH VERSION

I. Questionnaire – Part 1

Demographics

Age (years):

Gender:

- ◯ Female
- ◯ Male
- ◯ Other: ____

Highest degree/qualification:

Did you live most of the last two years (2020-2021) in Italy?

- ◯ Yes
- ◯ No

If so, please select the region and province you sent the most of your last two years:

Region:

Province:

Start of Block: Domande legate al benessere (ansia e depressione)

Did you ever suffer from neurological (head trauma, ictus) or psychiatric conditions (depression, personality disorders)?

- ○ Yes, neurological conditions
- ○ Yes, psychiatric conditions
- ○ No, never
- ○ Both

Please specify which condition:

- ▢ Ictus or other acquired cerebral lesions
- ▢ Parkinson’s Disease
- ▢ Alzheimer’s Disease
- ▢ Multiple Sclerosis
- ▢ Other (*specify*): ____

Please specify which condition:

- [ ] Mood Disorders (depression, bipolar disorder)
- [ ] Personality Disorders
- [ ] Anxiety Disorders
- [ ] Obsessive-Compulsive Disorder
- [ ] Psychotic Disorders
- [ ] Eating Disorders
- [ ] Other (*specify*): ____

Are you currently assuming psychiatric drugs?

○ Yes

○ No

The following questions refer to your level of well-being.
ISTRUZIONI: Please read each statement and indicate how often the statement applied to you over the past week. There are no right or wrong answers. Do not spend too much time on any statement.

	Never	Sometimes	Often	Always / Almost all the time
1. I found it hard to wind down	○	○	○	○
2. I was aware of dryness of my mouth	○	○	○	○
3. I couldn't seem to experience any positive feeling at all	○	○	○	○
4. I experienced breathing difficulty (e.g., excessively rapid breathing, breathlessness in the absence of physical exertion)	○	○	○	○
5. I found it difficult to work up the initiative to do things	○	○	○	○
6. I tended to over-react to situations	○	○	○	○
7. I experienced trembling (e.g., in the hands)	○	○	○	○
8. I felt that I was using a lot of nervous energy	○	○	○	○
9. I was worried about situations in which I might panic and make a fool of myself	○	○	○	○
10. I felt that I had nothing to look forward to	○	○	○	○

11. I found myself getting agitated	○	○	○	○
12. I found it difficult to relax	○	○	○	○
13. I felt downhearted and blue	○	○	○	○
14. I was intolerant of anything that kept me from getting on with what I was doing	○	○	○	○
15. I felt I was close to panic	○	○	○	○
16. I was unable to become enthusiastic about anything	○	○	○	○
17. I felt I wasn't worth much as a person	○	○	○	○
18. I felt that I was rather touchy	○	○	○	○
19. I was aware of the action of my heart in the absence of physical exertion (e.g., sense of heart rate increase, heart missing a beat)	○	○	○	○
20. I felt scared without any good reason	○	○	○	○
21. I felt that life was meaningless	○	○	○	○

II. TASK: Link to the Pavlovia Page

Start of Block: Link to the task (video inside)

Here I ask you to add an alphanumeric code (ID_Subject) through which your responses to the questionnaire and the experiment will be associated, in a totally anonymous way. The code, that you will insert at the beginning of the experimental task you will find through the following link, will be composed of the initial letters of your name and surname, your age, gender in which you identify yourself (please write F, M, or A for other) and the day of the month in which you are performing the experiment (es. Mario Rossi, age 23, gender M, experiement performed the 3rd of october: MR23M3).

Here’s the link to start the experiment:

https://run.pavlovia.org/Stefano_PhD/thai_nega/html

Remember to add the code you just created as your “ID_Subject”. Once the task is finished, come back to this page to answer to some final questions.

III. Questionnaire – Part 2

Experience with the pandemic

Have you ever contracted COVID-19?

○ YES

○ NO

Are you currently positive to COVID-19?

○ YES

○ NO

If so, which symptoms are/were associated with COVID-19? (*You can select multiple responses*)

- ☐ Fever
- ☐ Cough
- ☐ Breathing difficulties
- ☐ Sense of tiredness/weakness
- ☐ None of these symptoms

Have you had other symptoms associated with COVID-19? (*You can select multiple responses*)

- ☐ Soreness, muscle aches
- ☐ Nasal Congestion
- ☐ Runny nose
- ☐ Sore throat
- ☐ Diarrhea
- ☐ Difficulty to perceive smells
- ☐ Difficulty to perceive flavors
- ☐ Greater/Lesser tactile sensitivity
- ☐ Hearing difficulties
- ☐ Visual Difficulties (such as “seeing double”, red/dry eyes, frequent tearing)

☐ None of the above

In which situation are you, regarding the dispositions and closures related to the COVID-19 pandemic emergency?

○ No restrictions (white zone)

○ I can go outside, but with some limits/restrictions (yellow/orange zone)

○ I can go outside only if strictly necessary (red zone)

○ I cannot go outside (total isolation/ quarantine)

If you are in quarantine, how many days have you spent in this situation?

____

How scared are you of being infected with COVID-19?

- In no way
- A little
- Mildly
- I’m quite worried
- A lot

How often do you look for information about it?

- Never
- Rarely
- Once per week
- Several times per week
- Every day

Do you know someone that contracted COVID-19?

	YES	NO
Relative or partner		
Friend		
Acquaintance		

Do you know someone that died due to COVID-19?

	YES	NO
Relative or partner	○	○
Friend	○	○
Acquaintance	○	○

Have you been vaccinated against COVID-19?

- ○ Yes, I completed the first cycle and received the third dose as well
- ○ Yes, I completed the first cycle (two doses)
- ○ Yes, but I haven’t completed the cycle yet (one dose out of two)
- ○ No, but I am planning to do it
- ○ No, I will not do it

The following questions refer to your level of well-being

INSTRUCTIONS: Referring to the current situation, characterized by the COVID-19 epidemic and the social distancing measures put in place to contain it, indicate how you feel for each of the following dimensions.

	Not at all	A little	Mildly	Quite a lot	Extremely
1. Having repeated, disturbing, unwanted thoughts about this stressful experience	○	○	○	○	○
2. Having repeated, disturbing dreams related to this stressful experience	○	○	○	○	○
3. Feeling very upset	○	○	○	○	○
4. Having strong physical reactions thinking about this stressful experience (e.g., pounding heart, difficulty breathing)	○	○	○	○	○
5. Try to avoid thoughts and feelings related to this stressful experience	○	○	○	○	○
6. Having difficulty thinking about other aspects of this stressful situation	○	○	○	○	○
7. Having trouble concentrating	○	○	○	○	○

8. Having trouble falling asleep	○	○	○	○	○
9. Having a disturbed sleep	○	○	○	○	○

Page Break

The current pandemic situation has changed various aspects of our daily lives. How would you rate the impact of COVID-19 on the following aspects of your life?

Rate it on a scale ranging from 0 (no impact) to 5 (strong impact).

	No Impact					Strong Impact
	0	1	2	3	4	5
WORKING / ECONOMIC						
PHYSICAL HEALTH						
PSYCHOLOGICAL HEALTH						
FAMILY RELATIONS						
SENTIMENTAL RELATIONS						
SOCIAL RELATIONS						

Have you ever been followed on a psychological level before the pandemic?

- ◯ YES
- ◯ NO

Have you sought psychological help during the pandemic?

- ◯ YES
- ◯ NO

Would you like to seek psychological help in the future?

- ◯ YES
- ◯ NO
